# Synergistic Effects of Bioactive Glass on the Physicochemical Properties and In Vitro Bioactivity of 3D-Printed PCL Scaffolds

**DOI:** 10.3390/ma19091740

**Published:** 2026-04-24

**Authors:** Bo Yang, Runhua Wang, Guang Yang, Zejia Zhang, Xiaohong Chen

**Affiliations:** 1 School of Health Science and Engineering, University of Shanghai for Science and Technology, Shanghai 200093, China; 2 School of Materials and Chemistry, University of Shanghai for Science and Technology, Shanghai 200093, China

**Keywords:** 3D printing, bioactive glass, polymeric materials, bone scaffold

## Abstract

Polycaprolactone (PCL) is widely utilized in bone tissue engineering due to its excellent biocompatibility and processability; however, its inherent bioinertness and hydrophobicity significantly restrict its clinical osteogenic efficacy. To overcome these limitations, we incorporated sol–gel synthesized silicate-based bioactive glass (BG) into a PCL matrix and fabricated a series of composite scaffolds with varying BG contents via direct ink writing (DIW) 3D printing. Rheological characterization confirmed that all ink formulations exhibited shear-thinning behavior, with viscosity increasing monotonically with BG content. DSC analysis revealed that BG incorporation progressively reduced the crystallinity of PCL from 51.47% to 36.23%. We systematically evaluated the physicochemical properties, mechanical resilience, and in vitro degradation behavior of these scaffolds. The results indicated that BG incorporation significantly improved the surface hydrophilicity, with the contact angle decreasing from 104.8 ± 2.81° to 69.8 ± 2.91°. Furthermore, as the BG content increased, the porosity and mechanical strength exhibited an initial increase followed by a subsequent decrease, yet all values remained within the range of human cancellous bone. Notably, cellular assays revealed that the introduction of 58SBG enhanced cell–matrix interactions; the PCL/BG scaffolds promoted superior cell attachment and more extensive morphological spreading compared to pure PCL. Among all groups, the PCL/30BG composite scaffold demonstrated the most optimal balance of mechanical integrity and biological response. Consequently, the PCL/30BG scaffold developed in this study exhibits immense potential as a bone graft substitute, providing a promising approach for clinical bone defect repair strategies.

## 1. Introduction

Bone defects stem from complex and diverse etiologies. Although native bone tissue possesses a remarkable capacity for self-repair and remodeling, this innate ability is often insufficient when confronted with large-scale defects exceeding the critical size [1]. Consequently, clinical intervention is generally required. However, conventional treatment modalities face significant challenges. Autologous bone grafting, despite being the gold standard for bone defect treatment, is frequently hindered by donor-site morbidity, prolonged surgical times, and limited availability [2]. Similarly, the use of metallic implants presents several drawbacks, including corrosion, the release of toxic ions, limited biocompatibility, and a mismatch in elastic modulus compared to native bone tissue, which can ultimately lead to stress shielding and implant failure [3]. Furthermore, the application of exogenous growth factors (such as BMPs) is associated with undesirable outcomes, including immunogenic responses, exorbitant costs, and adverse side effects resulting from the required supraphysiological doses [4,5].

Therefore, an ideal bone substitute material must exhibit excellent biocompatibility, matched mechanical properties, robust bioactivity to stimulate osteoblast proliferation, and an appropriate degradation rate [6,7]. In this context, bone tissue engineering (BTE) offers a highly promising strategy for defect repair. Generally, this strategy relies on the classic tissue engineering triad: scaffolds, seed cells, and bioactive factors. Among these, the engineered scaffold serves as a three-dimensional (3D) template that replicates the structural and functional attributes of the native extracellular matrix (ECM), thereby facilitating cell attachment, proliferation, and subsequent tissue regeneration [8].

To effectively emulate natural bone, the majority of contemporary scaffolds are designed as polymer–ceramic composites [9]. In such systems, the polymeric matrix is formulated from natural or synthetic polymers, while the ceramic phase typically consists of bioactive glass (BG) and calcium phosphate (CaP) compounds, such as hydroxyapatite (HA) and biphasic calcium phosphates (BCPs) [10]. Extensive research has demonstrated that these composite scaffolds provide a reliable and effective solution for the regenerative repair and functional reconstruction of bone defects.

Polycaprolactone (PCL) is a semi-crystalline, linear aliphatic polyester that is synthesized from ε-caprolactone monomers via a ring-opening polymerization reaction [11]. Due to its numerous advantages, such as good tissue compatibility, biodegradability, favorable mechanical properties, and low cost, it has become a preferred material for research and development in bone tissue engineering. Furthermore, PCL’s high crystallinity, ease of processing, and excellent rheological and viscoelastic properties also endow it with outstanding printability [12,13]. It is worth mentioning that when PCL is implanted in the body, it can be ultimately converted into CO_2_ and H_2_O through enzymatic degradation. These byproducts can then be readily excreted from the body through its natural metabolic pathways [14]. However, a notable drawback is the slow degradation rate of PCL, which typically requires 2 to 3 years to degrade completely. This means that during the bone tissue repair process, the pure PCL material remains in the patient’s body for a prolonged period, which can hinder the natural remodeling and regeneration of bone tissue [15]. Furthermore, a key issue is that the hydrophobic nature of PCL can impair cell adhesion and proliferation [16]. Additionally, although it possesses a certain degree of mechanical strength, in load-bearing applications, pure PCL scaffolds often fail to provide sufficient mechanical support [17]. For these reasons, it is necessary to develop a more reliable and effective strategy to overcome the shortcomings of PCL material in order to enhance its performance in bone repair applications [18].

Due to its excellent bioactivity, biocompatibility, and osteoconductivity, bioactive glass (BG) has been widely applied in the field of bone tissue engineering (BTE) since its discovery over 50 years ago [19]. Incorporating BG into polymer matrices can overcome the inherent limitations of pure polymers, such as polycaprolactone (PCL), particularly its lack of osteoinductivity and biological inertness, while maintaining reliable mechanical properties suitable for load-bearing applications [20,21]. Among various bioactive glasses, 58S BG synthesized via the sol–gel method has attracted considerable attention. Compared to conventional melt-derived glasses, sol–gel-derived 58S BG possesses a highly porous network and a larger specific surface area, which endow it with superior in vitro bioactivity and a faster degradation rate [22,23]. Consequently, incorporating this highly reactive 58S BG into a slow-degrading PCL matrix serves multiple vital functions: it not only accelerates the deposition of a bone-like hydroxyapatite (HA) layer to rapidly promote damaged bone repair, but it also helps neutralize the acidic degradation products of PCL. Ultimately, this synergistic effect creates a highly favorable microenvironment for cell proliferation and osteogenesis [24,25].

In bone tissue engineering, among the various strategies for enhancing scaffold performance, the role of the architectural structure is of critical importance. It allows for control over key features such as porosity, mechanical properties, and complex scaffold geometries [26]. Notably, 3D printing technology meets these requirements, offering significant advantages such as short fabrication cycles, strong material compatibility, and the ability for personalized customization. It has shown enormous potential, particularly in the manufacturing of bone implants [27,28]. As an important branch of 3D printing, Direct Ink Writing (DIW) technology shows broad application prospects for constructing bone tissue engineering scaffolds with complex, biomimetic structures. This is due to its numerous advantages, including wide material adaptability, high resolution, excellent surface quality, controllable microstructure, and the absence of a need for high temperatures or high-energy input.

In this study, 58S bioactive glass (BG) was synthesized via a sol–gel method, and polycaprolactone/bioactive glass (PCL/BG) composite scaffolds were subsequently fabricated using direct ink writing (DIW) 3D printing technology. The composite scaffolds with varying BG ratios were systematically characterized to evaluate their surface morphology, elemental distribution, chemical structure, porosity, mechanical properties, water retention capacity, and surface hydrophilicity (contact angle). Furthermore, their in vitro bioactivity in simulated body fluid (SBF) and effects on early cell adhesion and morphological spreading were thoroughly investigated. This research highlights the immense potential of the composite material in synergistically enhancing mechanical strength and biological responses, providing valuable insights and a reliable reference for the future development of bone tissue engineering scaffolds.

## 2. Materials and Methods

### 2.1. Materials

Polycaprolactone (PCL, Mw = 80,000) was purchased from Sigma-Aldrich (Darmstadt, Germany). Hydrochloric acid (HCl, analytical grade) was obtained from Aladdin Biochemical Technology Co., Ltd. (Shanghai, China). Tetraethyl orthosilicate (TEOS), triethyl phosphate (TEP), calcium nitrate tetrahydrate (Ca(NO_3_)_2_·4H_2_O), and chloroform (CHCl_3_) of analytical grade were all purchased from Sinopharm Chemical Reagent Co., Ltd. (Shanghai, China).

For the preparation of simulated body fluid (SBF), sodium chloride (NaCl, analytical grade), potassium chloride (KCl, guaranteed reagent), anhydrous calcium chloride (CaCl_2_, analytical grade), magnesium chloride hexahydrate (MgCl_2_·6H_2_O, analytical grade), sodium bicarbonate (NaHCO_3_, analytical grade), dipotassium hydrogen phosphate trihydrate (K_2_HPO_4_·3H_2_O, analytical grade), sodium sulfate (Na_2_SO_4_, chemically pure), and Tris(hydroxymethyl)aminomethane (Tris, analytical grade) were also supplied by Sinopharm Chemical Reagent Co., Ltd. Deionized (DI) water with a resistivity of 18.2 MΩ·cm at 25 °C was produced using a Direct-Q5 UV water purification system (Shanghai Tianneng Technology Co., Ltd., Shanghai, China) and used throughout the experiments.

### 2.2. Synthesis and Characterization of 58S Bioactive Glass (BG)

58S bioactive glass (BG, composed of 58 mol% SiO_2_, 33 mol% CaO, and 9 mol% P_2_O_5_) was synthesized via the sol–gel method [29]. Initially, approximately 29.5g of tetraethyl orthosilicate (TEOS, Si(OC_2_H_5_)_4_) and a specific amount of hydrochloric acid were added to deionized water. The mixture was magnetically stirred in a sealed container for 30 min to ensure complete hydrolysis. Subsequently, 4.8 g of triethyl phosphate (TEP, OP(OC_2_H_5_)_3_) was introduced, and stirring was continued for 1 h. Upon the complete dissolution of TEP, 15.7 g of calcium nitrate tetrahydrate (Ca(NO_3_)_2_·4H_2_O) was added, and the resultant solution was stirred at room temperature for 6 h. Following this, the obtained sol was transferred to a beaker and aged for 72 h to facilitate hydrolysis and polycondensation reactions, yielding a wet gel. The bulk wet gel was then dried in an oven at 70 °C for 24 h to form a dry gel, which was subsequently heat-treated in a high-temperature furnace at 750 °C for 5 h. This process produced the bioactive glass as a white powder. Finally, the powder was ground and stored in a sealed container.

The surface morphology of the 58S bioactive glass (BG) powder was observed using a scanning electron microscope (SEM, Quanta 450, FEI, Hillsboro, OR, USA). Prior to the examination, the powder samples were sputter-coated with gold for 120 s to enhance conductivity. Additionally, the elemental distribution of silicon (Si), sodium (Na), calcium (Ca), and phosphorus (P) within the BG powder was analyzed using an energy-dispersive X-ray spectroscopy (EDS, Quanta 450, FEI, Hillsboro, OR, USA) detector attached to the SEM.

### 2.3. Fabrication and Characterization of PCL/BG Composite Scaffolds

To prepare the bio-ink for the composite scaffolds, 2 g of PCL pellets were dissolved in a solvent mixture of chloroform and dimethyl sulfoxide (DMSO) at a volume ratio of 6.8:0.2 (mL/mL) under magnetic stirring at 300 rpm for 2 h to obtain a homogeneous and stable solution. Subsequently, bioactive glass (BG) powder was added at varying mass fractions (0%, 10%, 20%, 30%, and 40%) and stirred thoroughly, as detailed in Table 1. The mixture was then continuously stirred in a fume hood to adjust its viscosity, yielding a bio-ink suitable for printing.

The rheological properties of the five composite ink formulations (Pure PCL, PCL/10%BG, PCL/20%BG, PCL/30%BG, and PCL/40%BG) were characterized using a TA HR-20 rotational rheometer (TA Instruments, New Castle, DE, USA) equipped with a parallel plate geometry (25 mm diameter). Prior to measurement, each ink sample was loaded onto the lower plate and allowed to equilibrate for 2 min at 25 °C to ensure thermal stability and eliminate residual stresses introduced during sample loading. Flow sweep tests were performed at 25 °C to obtain shear-rate–viscosity curves. The apparent viscosity was recorded as a function of shear rate ranging from 0.01 to 100 s^−1^ in logarithmic increments. Each measurement was performed in triplicate (*n* = 3) and the results were expressed as mean values.

**Figure 1:**
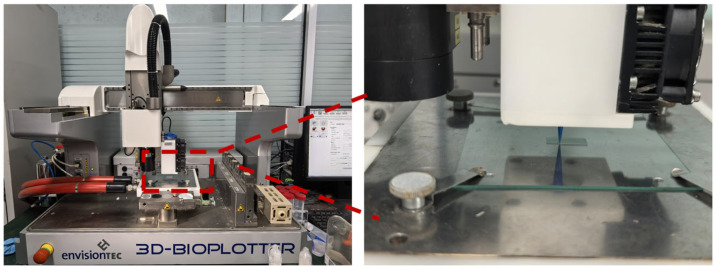
Schematic diagram of the 3D printing process.

**Figure 2:**
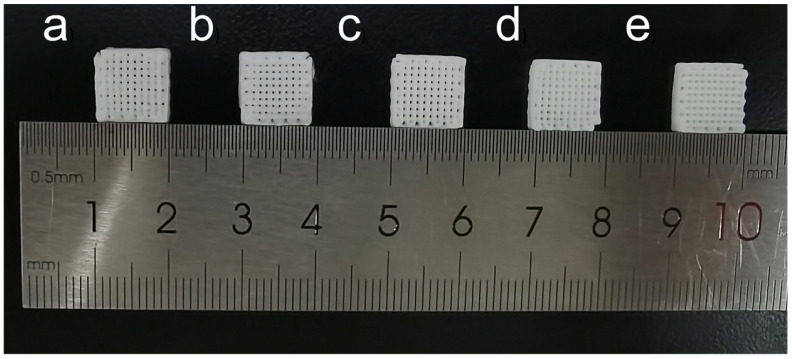
Digital photographs of the fabricated PCL/BG composite scaffolds. From left to right: (**a**) Pure PCL, (**b**) PCL/10%BG, (**c**) PCL/20%BG, (**d**) PCL/30%BG, and (**e**) PCL/40%BG.

The scaffold models were designed using Perfactory RP software (version 3.2, EnvisionTEC GmbH, Gladbeck, Germany). Next, the formulated bio-ink was transferred into a 30 cc syringe equipped with a dispensing nozzle at room temperature. The scaffolds were then extruded using a 3D-Bioplotter (EnvisionTEC, Gladbeck, Germany) through a nozzle with an inner diameter of 400 μm under an applied pressure of 3–5 × 10^5^ Pa, as illustrated in Figure 1. The X-Y platform speed was maintained at 5–15 mm/s, and the Z-axis layer height was set to 400 μm. In this study, orthogonal scaffolds with dimensions of 10 × 10 × 5 mm were fabricated. Following the printing process, all scaffolds were left at room temperature for 2 days to evaporate any residual solvents. The digital photographs of the fabricated scaffolds are presented in Figure 2, confirming the successful fabrication of orthogonal porous scaffolds with consistent dimensions across all groups.

Compressive strength tests were performed on the scaffold samples with dimensions of 10 × 10 × 5 mm using an electronic universal testing machine (Z2.-5TH, ZwickRoell, Ulm, Germany). During the test, the scaffold samples were longitudinally compressed at room temperature at a loading rate of 0.5 mm/min until the strain reached 60%. For each test, a minimum of six samples were used.

The porosity of the PCL/BG scaffold samples was determined using the pycnometer method, which is based on Archimedes’ principle [30]. Deionized water was used as the liquid medium. Prior to measurement, all scaffold samples were immersed in deionized water and subjected to vacuum degassing for 15 min to ensure complete infiltration of water into the pore structure. The porosity of the scaffold samples (*n* = 3) was calculated according to the following formula, and the average value of all recorded results was determined:

$$P(\%)=\frac{W_{2}-W_{3}-W_{0}}{W_{1}-W_{3}}\times 100\%$$

where *W*_0_ represents the dry weight of the scaffold in air; *W*_1_ is the weight of the pycnometer filled with deionized water; *W*_2_ corresponds to the weight of the pycnometer containing the scaffold and filled with deionized water; and W_3_ is the weight of the pycnometer and the remaining deionized water after the scaffold is removed.

The surface hydrophilicity of the composite scaffold samples with varying BG content was assessed using the contact angle method. The measurements were conducted at room temperature using the static sessile drop technique with an interface tension tester (JC2000C1, Shanghai Zhongchen Digital Technology Equipment Co., Ltd., Shanghai, China). First, a sample with a flat and clean surface was placed on a horizontal test plate and secured to prevent movement during the test. Then, a microsyringe filled with deionized water was used to manually and slowly deposit a water droplet onto three different locations on each scaffold. Finally, a high-definition camera monitored the water droplet on the sample surface in real-time. The contact angle was measured and recorded once the droplet reached a stable state.

To evaluate the stability and adaptability of the scaffold samples in a liquid environment, a water retention rate test was conducted. Before the test, the printed scaffold samples were pre-treated in a freeze-dryer (SCIENTZ-25T, Ningbo Scientz Biotechnology Co., Ltd., Ningbo, China) for 12 h. After this treatment, the weight of each scaffold sample was measured using an electronic balance (AUW220D, Shimadzu Global Lab Consumables Co., Ltd., Shanghai, China). Subsequently, the samples were individually placed in separate polyethylene bottles (150 mL) containing 100 mL of phosphate-buffered saline (PBS) solution. The bottles were then transferred to a thermostatic water bath shaker (SHA-AB, Shanghai Canjing Instrument Co., Ltd., Shanghai, China), with the water temperature set to 37 °C and a shaking speed of 120 rpm. After 1, 2, 4, 8, 12, 18, 24, 28, 32 and 36 h of incubation, the samples were removed, the liquid attached to the surface was quickly wiped away with filter paper, and the wet weight of each scaffold sample was measured and recorded. The water retention rate was calculated using the following formula, and the average of all recorded results was obtained:

$$\mathrm{Water}\;\mathrm{retention}\;\mathrm{rate}(\%)=\frac{W_{wet}-W_{dry}}{W_{dry}}\times 100\%$$

where *W_dry_* is the initial dry weight of the scaffold sample before immersion and *W_wet_* is the wet weight of the scaffold sample after immersion at different time points.

To evaluate the chemical composition of the scaffolds, attenuated total reflectance Fourier transform infrared (ATR-FTIR) spectroscopy was employed. Prior to the measurement, the respective scaffold samples were pulverized into fine powders. The analysis was conducted utilizing a Fourier Transform Infrared spectrometer (M6RE0134, Tianjin Gangdong Sci & Tech Co., Ltd., Tianjin, China). The chemical structures were characterized by acquiring all spectra in the range of 4000–600 cm^−1^ at a resolution of 4 cm^−1^, with each final spectrum representing the average of 64 consecutive scans.

The thermal properties and crystallinity of the PCL/BG composite scaffolds were characterized by differential scanning calorimetry (DSC) using a DSC 8000 instrument (PerkinElmer, Waltham, MA, USA) under a nitrogen atmosphere. Each sample (3–5 mg) was placed in an aluminum crucible and heated from 25 °C to 100 °C at a heating rate of 10 °C/min. The melting enthalpy (ΔHm) of each sample was determined by integrating the area under the melting peak using the Pyris software (version 8.16.0, PerkinElmer, Waltham, MA, USA). The degree of crystallinity (Xc) of the PCL phase in each composite was calculated according to the following equation:

$$x_{c}\left(\%\right)=\frac{∆H_{m}}{\left(\Delta {\mathrm{Hm}}^{{}^{\circ}}\times wPCL\right)}\times 100\%$$

where ΔHm is the measured melting enthalpy of the sample (J/g), ΔHm° = 139.5 J/g is the theoretical melting enthalpy of 100% crystalline PCL, and wPCL is the weight fraction of PCL in the composite (i.e., 1.0, 0.9, 0.8, 0.7, and 0.6 for Pure PCL, PCL/10%BG, PCL/20%BG, PCL/30%BG, and PCL/40%BG, respectively).

The crystalline structures of the specimens before and after SBF incubation were assessed using a Bruker D8 ADVANCE X-ray diffractometer (Bruker AXS GmbH, Karlsruhe, Germany) utilizing Cu Kα radiation (40 kV, λ = 0.154 nm). The continuous scanning was performed across a 2θ range of 10–70° at a set speed of 10°/min. Finally, the generated spectra were evaluated using OriginPro 2021 (version 9.8.0.200, Corporation, Northampton, MA, USA)to confirm the deposition of hydroxyapatite.

To evaluate the ability of the scaffolds to form mineralized hydroxyapatite and to assess their dissolution, the changes in surface morphology before and after SBF immersion were observed using SEM. Notably, due to the poor conductivity of the samples, the scaffolds were mounted on stubs with conductive adhesive and sputter-coated with gold for 100 s using a coating machine (Q150TES, Quorum, Laughton, UK) before imaging to ensure clear observation.

The degradation characteristics of the scaffold samples were determined by the weight loss method. Before immersion, the initial dry weight of each scaffold sample was accurately weighed and recorded. The samples were removed after 0, 2, 4, and 6 weeks of immersion. It is noteworthy that to prevent contamination, the SBF solution was replaced every 48 h. After removal, the samples were rinsed three times with deionized water and freeze-dried for 12 h. The samples were weighed again, and the residual mass of the scaffold samples was calculated according to the following formula:

$$\mathrm{Residual}\;\mathrm{mass}(\%)=\frac{W_{a}}{W_{b}}\times 100\%$$

where *W_a_* is the weight of the scaffold sample after immersion in SBF at a specific time point, and *W_b_* is the weight of the scaffold sample before immersion.

### 2.4. Scaffold In Vitro Degradation

Scaffold samples from each group were placed in polyethylene bottles (150 mL) containing 100 mL of SBF. The simulated body fluid (SBF) was prepared according to the method described by Kokubo [31]. The bottles were then incubated in a thermostatic water bath shaker at 37 °C and 120 rpm. The scaffolds were collected after 2, 4, and 6 weeks of incubation, during which the SBF solution was refreshed every two days. Subsequently, the collected scaffolds were rinsed three times with deionized water and dried in a freeze-dryer for 12 h. At predetermined time intervals of 2, 4, and 6 weeks, the samples were retrieved from the SBF solution and stored in a desiccator for subsequent scanning electron microscopy (SEM, Quanta 450, FEI, Hillsboro, OR, USA), X-ray diffraction (XRD, Bruker AXS GmbH, Karlsruhe, Germany), and biodegradability analyses.

### 2.5. Cell Experiment

Prior to cell culture, the five types of scaffolds were cut to dimensions of 5 mm × 5 mm × 2 mm and immersed in 75% (*v*/*v*) ethanol. After removal, the samples were air-dried and sterilized under ultraviolet (UV) irradiation for 2 h. The sterilized scaffolds were then placed into a 96-well plate. MC3T3-E1 cells (Shanghai Meiyan Biotechnology Co., Ltd., Shanghai, China) were seeded onto the scaffolds at a density of 5 × 10^4^ cells/well in a volume of 100 μL per well. Three replicate wells were set up for each group.

The specimens were maintained in a 37 °C, 5% CO_2_-humidified environment for 24 h. Upon completion of the culture phase, the liquid medium was removed, and a triple PBS rinse was applied to clear away non-adherent cells. For fixation, cells were exposed to 4% paraformaldehyde (PFA) for 20 min at room temperature, followed by three rounds of PBS washing. Cellular permeabilization was then conducted using 0.1–0.5% Triton X-100 in PBS for 5–10 min at room temperature, concluding with another three PBS rinses.

Regarding fluorescence labeling, the samples were incubated with an FITC-phalloidin working solution for 30 min in the absence of light to stain the cytoskeleton. Unbound dye was washed away with PBS, and DAPI was subsequently applied for 5 min in the dark to highlight the cell nuclei. After a final wash, the prepared samples were placed on glass slides for imaging with a Carl Zeiss LSM 710 confocal laser scanning microscope (Carl Zeiss AG, Jena, Germany).

All data are expressed as the mean ± standard deviation (SD). Statistical analysis was performed using one-way analysis of variance (ANOVA). A value of *p* < 0.05 was considered statistically significant. Statistically significant differences are indicated by different lowercase letters.

## 3. Results and Discussion

### 3.1. Morphology and Phase Analysis of BG

**Figure 3:**
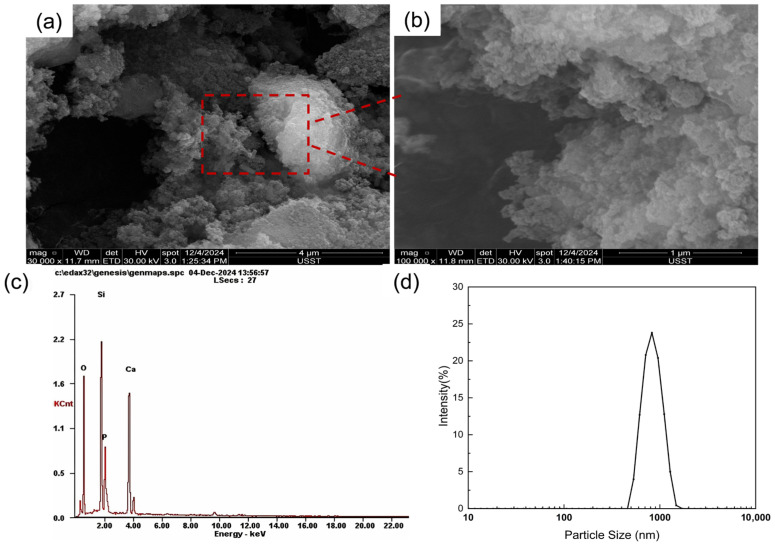
Morphology and phase analysis of BG: (**a**) general morphology (30,000×); (**b**) magnified view of the local surface (100,000×); (**c**) EDS spectrum and elemental composition of the sample; (**d**) DLS size distribution of the 58S BG powder suspended in ethanol.

The 58S bioactive glass (BG) powder was successfully synthesized via the sol–gel method. As shown in Figure 3a, scanning electron microscopy (SEM) at a magnification of 30,000× reveals that the BG particles exhibit a typical irregular polyhedral shape, with a certain degree of physical agglomeration. Further observation at a higher magnification (Figure 3b) demonstrates that the surface of these micron-sized particles is essentially a porous structure composed of dense nanoscale subunits. This unique nanoporous structure, a hallmark of the sol–gel process, endows the material with a remarkably high specific surface area, which is highly favorable for rapid ion exchange and the subsequent formation of a bone-like hydroxyapatite (HA) layer in a physiological environment [23,32].

Furthermore, the energy-dispersive X-ray spectroscopy (EDS) profile (Figure 3c) clearly displays the characteristic peaks of Si, Ca, P, and O. Importantly, the stoichiometric Ca/P ratio is very close to 1.67, aligning well with the standard composition of 58S bioactive glass. The particle size distribution (Figure 3d) exhibits a distinct unimodal distribution with a narrow peak centered approximately between 800 and 900 nm, indicating excellent dispersibility of the BG powder within the printing ink [33].

### 3.2. Ink Printability Evaluation

**Figure 4:**
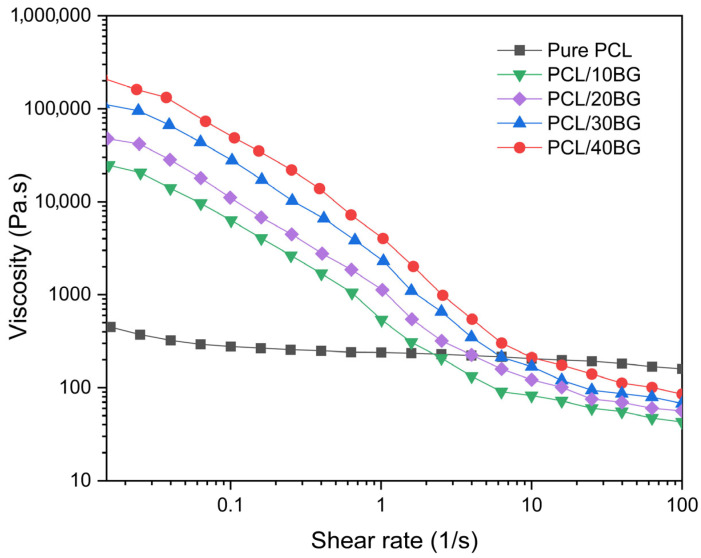
Shear-rate–viscosity curves of Pure PCL and PCL/BG composite inks with varying BG content (10, 20, 30, and 40 wt%). All measurements were performed at 25 °C using a TA HR-20 rotational rheometer with parallel plate geometry.

The shear rate–viscosity curves of all five composite ink formulations are presented in Figure 4. All BG-containing formulations exhibited shear-thinning (pseudoplastic) behavior, with apparent viscosity decreasing with increasing shear rate over the measured range (0.03–100 s^−1^). In contrast, Pure PCL exhibited a relatively flat viscosity profile with negligible shear-thinning behavior.

At low shear rates, the apparent viscosity increased with BG content, from approximately 500 Pa·s for Pure PCL to approximately 20,000, 50,000, 100,000, and 200,000 Pa·s for PCL/10%BG, PCL/20%BG, PCL/30%BG, and PCL/40%BG, respectively. This viscosity increase is attributed to the role of BG particles as rigid fillers, which generate internal friction within the PCL matrix and elevate the apparent viscosity of the composite ink [34].

The shear-thinning behavior observed in all BG-containing formulations confirms their suitability for extrusion-based 3D printing, as the reduction in viscosity under applied shear facilitates material flow through the nozzle [35]. Based on these rheological profiles, the extrusion pressure and printing speed were adjusted for each formulation to achieve consistent filament deposition.

### 3.3. Analysis of Mechanical Properties of PCL/BG Composite Scaffolds

**Figure 5:**
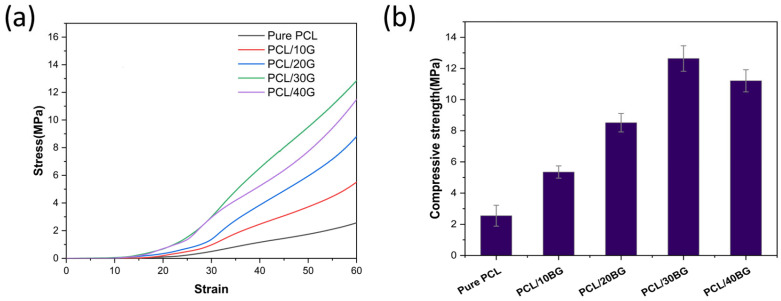
Mechanical properties of the PCL/BG composite scaffolds: (**a**) Compressive stress–strain curves; (**b**) Compressive strength.

Figure 5 illustrates the mechanical properties of the composite scaffolds. Compared to the pure PCL group (3.55 ± 0.67 MPa), the incorporation of bioactive glass (BG) powder led to a significant increase in compressive strength. Specifically, the compressive strengths of the PCL/10BG, PCL/20BG, PCL/30BG, and PCL/40BG groups were measured at 5.35 ± 0.39, 8.52 ± 0.59, 12.64 ± 0.82, and 11.21 ± 0.71 MPa, respectively.

This mechanical enhancement can be attributed to the effective load transfer mechanism. When the composite material is subjected to compressive stress, a substantial portion of the load is transferred from the flexible PCL matrix to the rigid BG particles. These stiff inorganic particles serve as the primary load-bearing phase, thereby restricting the deformation and failure of the PCL molecular chains [36].

Notably, this reinforcing effect exhibits a threshold. An excessive BG content, as observed in the PCL/40BG group, can induce particle agglomeration. These agglomerates act as deleterious stress concentration sites. Simultaneously, the polymer matrix may fail to effectively encapsulate the excessive particles, leading to weakened interfacial adhesion and a subsequent decline in mechanical performance [36,37].

### 3.4. Porosity Analysis of the Composite Scaffolds

According to the literature, trabecular bone exhibits a natural porosity of 50–90%, and scaffolds with similarly high porosity are generally recommended for bone regeneration [38]. High porosity creates a large, interconnected network of pores within the scaffold, which facilitates nutrient transport, vascularization, and the ingrowth of osteoblasts, ultimately promoting the formation of new bone tissue [38].

**Figure 6:**
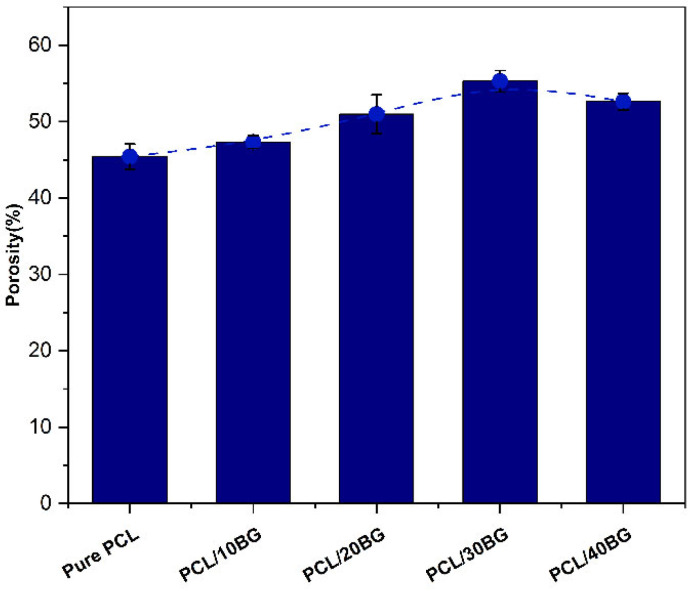
Porosity of the PCL/BG composite scaffolds.

As shown in Figure 6, it can be observed that when the addition of BG powder was relatively low (≤30 wt.%), the porosity of the scaffold samples gradually increased with the powder content. The porosity of the PCL/30BG group reached as high as 55.29 ± 1.41%, which is nearly 21.7% higher than that of the pure PCL group. This is because the BG particles, acting as rigid fillers, generate significant internal friction within the PCL, markedly increasing the apparent viscosity of the composite ink. Inks with higher viscosity exhibit a more pronounced die swell effect upon extrusion. The greater the increase in viscosity, the more significant the die swell effect, which systematically increases the microporosity [34,35,39].

However, when the BG content reached 40%, the excess BG particles began to contact each other, forming a continuous three-dimensional particle network. The PCL melt, in turn, became partitioned and entrapped, acting as a dispersed phase filling the gaps between the particles. The rheological behavior of the material shifted towards that of a polymer–powder paste. Consequently, the die swell effect was greatly diminished or even eliminated. There was almost no elastic recovery after extrusion, and the filament diameter was closer to, or even smaller than, the nozzle orifice. The structure formed by the stacking of these more rigid filaments was therefore denser, leading to a decrease in macroporosity.

### 3.5. Hydrophilicity Analysis of the Composite Scaffolds

**Figure 7:**
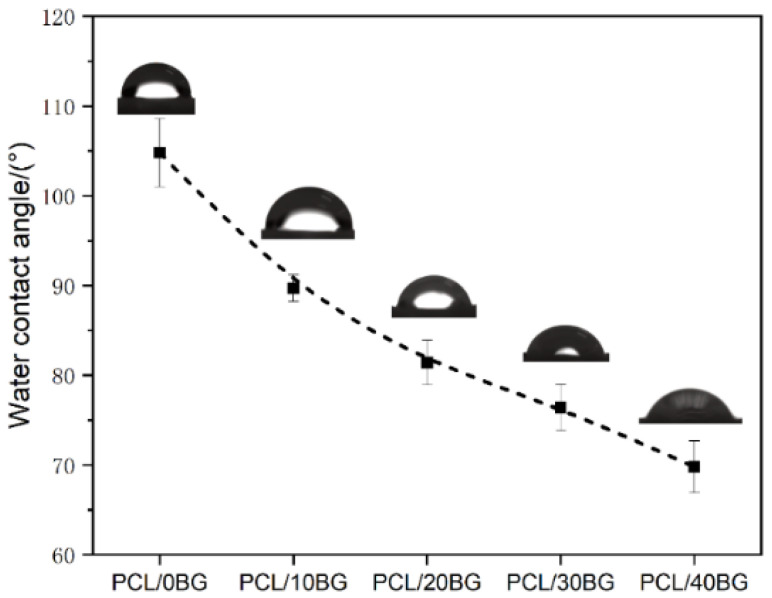
Contact angle of the PCL/BG composite scaffolds.

As shown in Figure 7, among the five scaffold sample groups, the pure PCL group exhibited the highest contact angle, at 104.8 ± 2.81°. As the content of BG powder in the scaffolds increased, the contact angle of the samples progressively decreased. The contact angles for the PCL/10BG, PCL/20BG, PCL/30BG, and PCL/40BG groups were 89.7 ± 1.47°, 81.4 ± 2.49°, 76.4 ± 2.6°, and 69.8 ± 2.91°, respectively, representing a maximum decrease of approximately 33.4%.

This trend is primarily due to the surface of pure PCL, which is mainly composed of nonpolar methylene chains (-CH_2_-) and weakly polar ester groups (-COO-), giving it an overall hydrophobic character and a large contact angle [40]. In contrast, the surface of BG particles is rich in numerous polar hydrophilic groups, such as silanol groups (-SiOH) [41]. When these particles are exposed on the composite’s surface, they create localized hydrophilic sites. Water molecules preferentially interact with these high-surface-energy sites via strong hydrogen bonding and spread across the surface, which significantly lowers the apparent contact angle of the entire material [42].

### 3.6. Water Retention Analysis of the Composite Scaffolds

**Figure 8:**
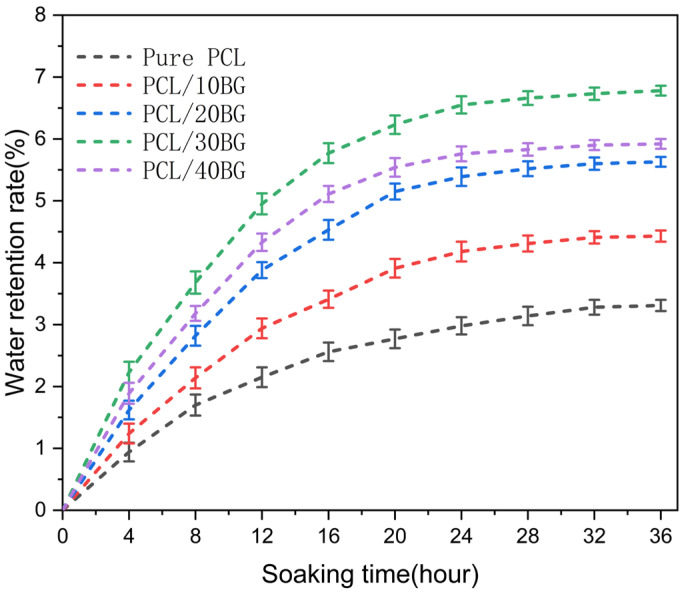
Water retention rate of the PCL/BG composite scaffolds. Data are presented as mean ± standard deviation (*n* = 3).

Figure 8 shows the water retention rate of the PCL/BG composite scaffolds after immersion in PBS for up to 36 h, during which all groups progressively reached swelling equilibrium after approximately 28 h. The pure PCL group exhibited the lowest water retention rate, at only 3.31 ± 0.09%, which can be attributed to its hydrophobic nature and small and dense pores, leading to low water permeability. After the introduction of BG powder into the PCL matrix, the pore structure and the surface area for water adsorption of the scaffolds increased, thereby enhancing their permeability and water retention rate. The PCL/30BG group reached a maximum water retention rate of 6.78 ± 0.08% after 36 h of immersion.

There are two main reasons for the subsequent decrease in the water retention rate for the PCL/40BG group (5.92 ± 0.08%). On one hand, the excessive amount of BG powder occupies more space within the PCL matrix, restricting the spatial arrangement and mobility of the PCL molecular chains. On the other hand, a large amount of BG powder in the PCL matrix can easily lead to interfacial defects and structural inhomogeneities within the scaffold, causing non-uniform localized swelling.

### 3.7. Structural Analysis of the Pre-Mineralized Composite Scaffolds

**Figure 9:**
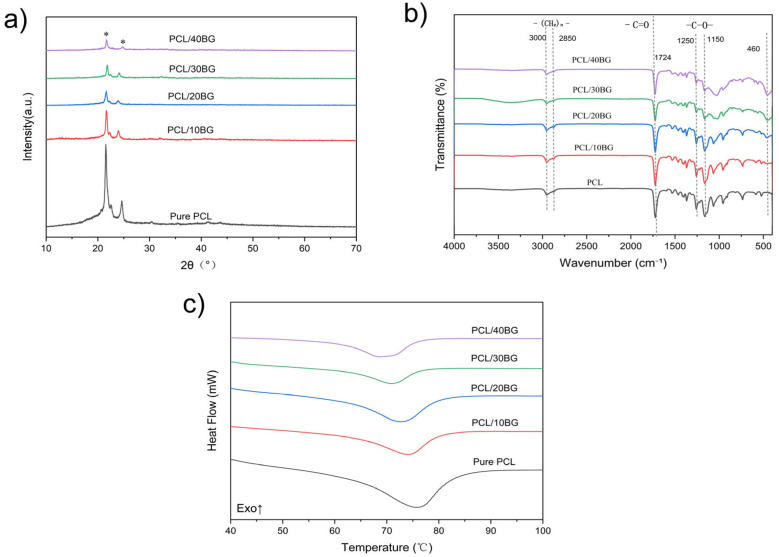
Structural analysis of the PCL/BG composite scaffolds: (**a**) XRD patterns; (**b**) FT-IR spectra; (**c**) DSC heating curves. (Note: All characterizations in this figure were performed on the scaffolds prior to any in vitro immersion). In (**a**), * denotes the characteristic diffraction peaks of the PCL crystalline phase at 2θ ≈ 21.4° and 23.7°, corresponding to the (110) and (200) crystallographic planes, respectively. In (**c**), “Exo↑” indicates the exothermic direction of heat flow.

The crystalline and chemical structures of the composite scaffolds were systematically investigated using X-ray diffraction (XRD), Fourier Transform Infrared Spectroscopy (FT-IR), and Differential Scanning Calorimetry (DSC), as illustrated in Figure 9. As shown in the XRD patterns (Figure 9a), the pure PCL scaffold exhibits two distinct diffraction peaks at approximately 2θ = 21.4° and 23.7°, corresponding to the (110) and (200) crystallographic planes of semi-crystalline PCL. Upon incorporation of 58S bioactive glass (BG), no new crystalline peaks emerged, which is consistent with the amorphous nature of BG. As the BG content progressively increased from 10 wt% to 40 wt%, the intensity of the characteristic PCL diffraction peaks gradually decreased, which may reflect the reduced fraction of crystalline PCL in the composite.

To further clarify whether the decrease in XRD peak intensity reflects a genuine reduction in PCL crystallinity rather than a simple dilution effect, DSC analysis was performed on all groups (Figure 9c). As summarized in Table 2, the melting enthalpy decreased systematically from 71.80 J/g for Pure PCL to 30.33 J/g for PCL/40%BG. After normalizing for the actual PCL weight fraction in each composite, the degree of crystallinity decreased from 51.47% for Pure PCL to 36.23% for PCL/40%BG. This confirms that the reduction in crystallinity reflects a genuine disruption of PCL chain ordering, rather than a simple dilution effect. Specifically, BG particles physically interfere with the mobility and rearrangement of PCL molecular chains during solidification, hindering the formation of ordered crystalline domains and thereby reducing the overall degree of crystallinity of the composite scaffolds.

This physical compounding behavior was further corroborated by FT-IR spectral analysis (Figure 9b). The pure PCL displays characteristic absorption peaks at ~3000 cm^−1^ and ~2850 cm^−1^ (–(CH_2_)_n_– stretching vibrations), ~1724 cm^−1^ (C=O stretching vibration of the ester group), and ~1250 cm^−1^ and ~1150 cm^−1^ (C–O stretching vibrations). Following the addition of BG, a new absorption peak appeared at ~460 cm^−1^, assigned to the Si–O–Si bending vibration of the BG network, and its intensity became progressively more pronounced with increasing BG content. Importantly, no new peaks or peak shifts were observed in the composite spectra compared to pure PCL, indicating that BG and PCL interact physically rather than chemically. Collectively, the combined XRD, FT-IR, and DSC results confirm the successful physical incorporation of BG into the PCL matrix without chemical reaction between the two components.

### 3.8. Analysis of the Appearance and Morphology of Composite Scaffolds

**Figure 10:**
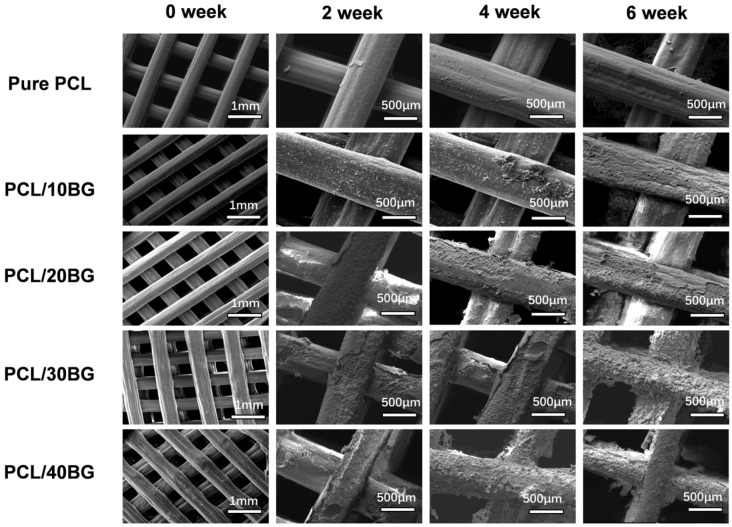
SEM micrographs of the PCL/BG composite scaffold samples before and after immersion in SBF solution for 0, 2, 4, and 6 weeks.

As illustrated in the scanning electron microscopy (SEM) images (Figure 10), to comprehensively evaluate the macroscopic printing fidelity and overall structural integrity of the scaffolds, the week 0 (pre-mineralization) samples were observed at a low magnification (1 mm scale bar). The results revealed that the as-printed scaffolds exhibited a well-defined, grid-like architecture with uniform strut diameters and relatively smooth surfaces. ImageJ analysis confirmed that the macropore sizes consistently exceeded 300 μm, a dimension highly sufficient to support subsequent cellular infiltration and internal vascularization [38]. Notably, the incorporation of BG powder resulted in a more granular and increasingly irregular strut surface. This morphological alteration is primarily attributed to the addition of BG, which increased the viscosity of the composite ink and compromised its printability. Furthermore, the potential presence of inadequately dispersed, excessive BG particles could easily induce localized stress concentrations during the extrusion process, leading to non-uniform strut diameters and surface wrinkling.

During the SBF immersion test, all scaffold formulations maintained their structural integrity without noticeable macroscopic collapse, perfectly preserving their highly interconnected porous networks. To deeply investigate the micro-mineralization mechanisms and localized degradation characteristics in SBF, a higher magnification (400 μm scale bar) was specifically employed to focus on the samples immersed for 2, 4, and 6 weeks. High-magnification SEM results demonstrated that after a 6-week immersion, the surface of the pure PCL scaffolds remained predominantly smooth, devoid of obvious degradation or peeling. This is largely restricted by the inherent bioinertness of PCL, which hinders its surface from effectively adsorbing Ca^2+^ and PO_4_^3−^ ions from the SBF solution, thereby impeding the nucleation and deposition of bone-like hydroxyapatite (HA) [43]. In stark contrast, the BG-incorporated composite scaffolds exhibited substantial deposition of micrometer-scale HA crystals on their surfaces, accompanied by the emergence of microcracks; moreover, this in vitro degradation and mineralization effect became increasingly pronounced with higher BG contents [44]. Nevertheless, despite the noticeable surface exfoliation exposing the underlying matrix after 6 weeks, none of the composite scaffolds experienced catastrophic fracture, fully verifying their excellent structural stability during the degradation process.

### 3.9. Analysis of the Hydroxyapatite Formation Process in SBF Solution

**Figure 11:**
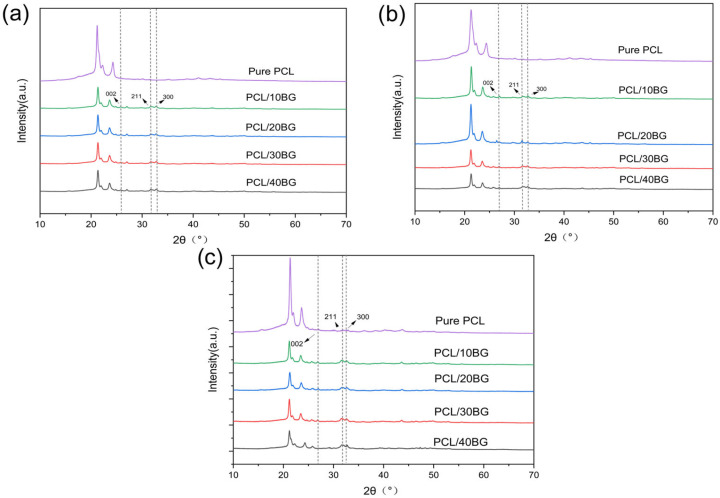
XRD patterns of the composite scaffolds after immersion: (**a**) 2 weeks, (**b**) 4 weeks, (**c**) 6 weeks.

As shown in Figure 11, after two weeks of immersion in SBF solution, new diffraction peaks appeared at 2θ = 32°, 32.9°, and 25.9° in the scaffolds containing BG. According to the JCPDS card, these peaks correspond to the (211), (300), and (002) crystal planes of HA, which is attributed to the gradual dissolution of BG and its subsequent reaction to form HA in the SBF solution. As the immersion time was extended from one to six weeks, the intensity of the characteristic peak for the HA (211) plane at 32° was slightly enhanced. This change indicates that upon contact with the SBF, ions such as Na^+^ and Ca^2+^ are rapidly released, leading to a local increase in their concentration. Concurrently, a highly active silanol (Si-OH) structure forms on the surface. The polymerization of these silanol groups results in the formation of a silica-rich gel layer, which serves as an ideal nucleation site for calcium and phosphate ions. This gel layer adsorbs Ca^2+^ and PO_4_^3−^ ions from the SBF, forming an amorphous calcium phosphate that subsequently crystallizes rapidly into a bone-like hydroxycarbonate apatite.

### 3.10. Analysis of the Degradation Performance of the Composite Scaffolds

**Figure 12:**
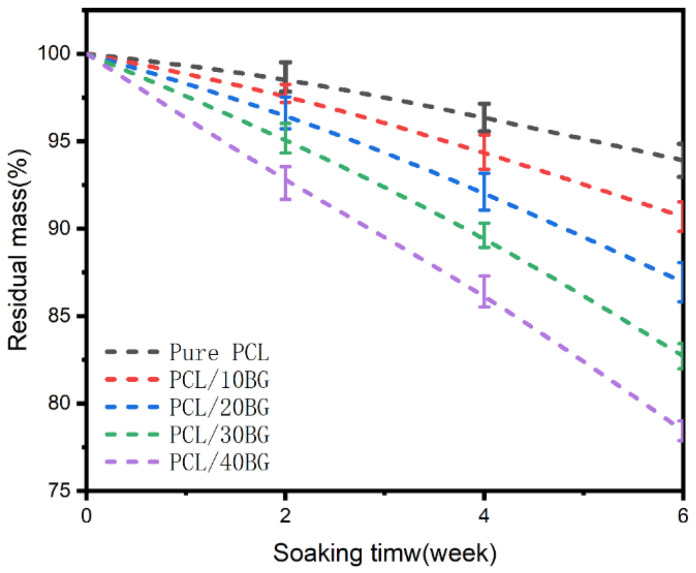
Residual mass of the PCL/BG composite scaffolds after incubation in SBF solution for 2, 4, and 6 weeks.

As shown in Figure 12, with increasing immersion time, the residual mass of all samples exhibited a downward trend, indicating that all scaffold materials underwent a certain degree of degradation in the SBF solution. The mass loss of pure PCL was the slowest. This is because the chemical structure of PCL is relatively stable and the hydrolysis rate of its ester bonds is slow; therefore, for the same immersion period, its mass loss was less, and its residual mass was higher.

As the bioactive glass content increased, the rate of residual mass loss for the scaffolds gradually accelerated. The PCL/40BG group experienced approximately 16.9% more mass loss than the pure PCL group. There are two potential reasons for this. On one hand, the interface between BG and PCL may act as an initiation site for degradation, making the ester bonds of PCL more susceptible to attack by water molecules from the SBF solution, thus accelerating hydrolysis. On the other hand, the ions released from the dissolution of BG could alter the chemical environment of the surrounding solution (e.g., pH value), which may further promote the degradation of PCL.

### 3.11. Effect of BG Content on Early Cell Adhesion and Morphology

**Figure 13:**
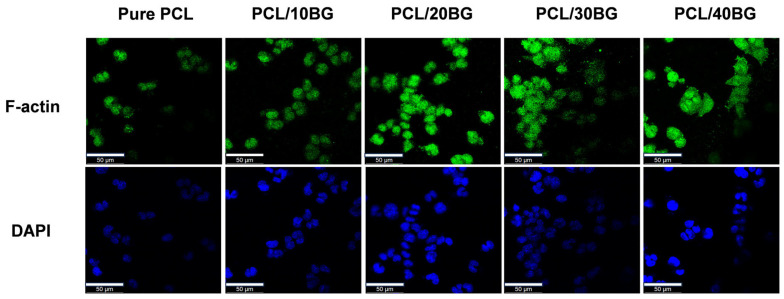
Early adhesion and morphological observation of MC3T3-E1 cells cultured on PCL/BG composite scaffolds with varying BG contents (0, 10, 20, 30, and 40 wt%) for 24 h. The cytoskeletal F-actin and cell nuclei were stained with FITC-phalloidin (green) and DAPI (blue), respectively. As the BG content increases, the cells exhibit a distinct morphological transition from adequate individual cell spreading to localized micro-agglomeration.

**Figure 14:**
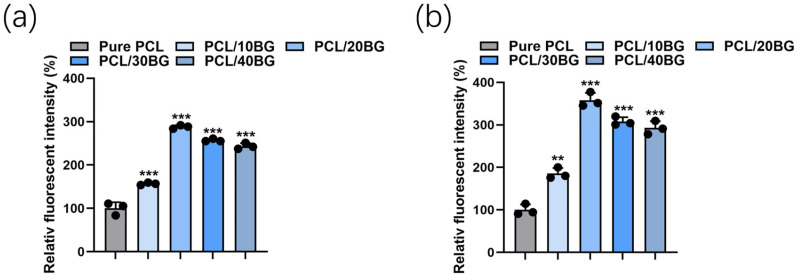
Quantitative fluorescence intensity analysis of MC3T3-E1 cells cultured on PCL/BG composite scaffolds with varying BG content after 24 h of culture. (**a**) Relative F-actin fluorescence intensity reflecting cytoskeletal development and cell spreading; (**b**) Relative DAPI fluorescence intensity reflecting the number of adherent cells. Pure PCL was set as the reference group (100%). Data are presented as mean ± SD (*n* = 3). ** *p* < 0.01, *** *p* < 0.001 vs. Pure PCL (one-way ANOVA with Tukey’s post hoc test).

The early adhesion and morphological evolution of MC3T3-E1 cells on 3D-printed scaffolds with varying BG gradients were evaluated using F-actin and DAPI staining after 24 h of culture (Figure 13). Quantitative analysis of relative fluorescence intensity was performed on both F-actin and DAPI-stained images using ImageJ software (version 1.54p, National Institutes of Health, Bethesda, MD, USA; https://imagej.org, accessed on 14 January 2026), with Pure PCL set as the reference group (100%). Statistical differences were assessed by one-way ANOVA with Tukey’s post hoc test (Figure 14).

On the pure PCL scaffolds, cells exhibited a restricted, spherical morphology with limited cytoskeletal spreading and obvious agglomeration, which is primarily attributed to the inherent hydrophobicity and the lack of bioactive recognition sites of the pristine PCL material [45].

With the incorporation of 10 wt% and 20 wt% BG, a noticeable improvement in cell spreading was observed. The relative F-actin fluorescence intensity of the PCL/10%BG and PCL/20%BG groups increased to approximately 160% and 290% of the Pure PCL group, respectively (*p* < 0.001), reflecting enhanced cytoskeletal development and cell spreading. Correspondingly, the relative DAPI fluorescence intensity increased to approximately 185% and 355%, respectively (*p* < 0.01 and *p* < 0.001), indicating a significantly greater number of adherent cells. These improvements can be attributed to the enhanced surface hydrophilicity and the moderate release of bioactive ions (Si, Ca) that effectively promoted integrin-mediated initial adhesion [46].

As the BG content further increased to 30 wt% and 40 wt%, both F-actin and DAPI fluorescence intensities remained significantly higher than that of the Pure PCL group (*p* < 0.001), with relative F-actin fluorescence intensities of approximately 260% and 245%, and relative DAPI fluorescence intensities of approximately 305% and 290%, respectively. However, a moderate decrease compared to the PCL/20%BG group was observed, which may be associated with surface heterogeneity resulting from localized BG particle agglomeration at elevated filler loadings [47].

Overall, all BG-containing groups demonstrated significantly enhanced cell adhesion relative to pure PCL, confirming the favorable biocompatibility of the composite scaffolds and the positive role of BG incorporation in promoting cell–material interactions.

## 4. Conclusions

In this study, a composite bio-ink with polycaprolactone (PCL) as the matrix and 58S bioactive glass (BG) as the functional filler was successfully formulated, and porous scaffolds with precisely controllable structures were fabricated using 3D printing technology. Rheological characterization confirmed that all composite ink formulations exhibited shear-thinning behavior, with apparent viscosity increasing monotonically with BG content, providing a rational basis for the selection of printing parameters. Systematic characterization results demonstrated that the addition of BG significantly improved the surface hydrophilicity of the scaffolds, indicating a higher potential for cell affinity. DSC analysis revealed that BG incorporation progressively reduced the crystallinity of PCL from 51.47% (Pure PCL) to 36.23% (PCL/40%BG), attributed to BG particles physically interfering with the rearrangement of PCL molecular chains during crystallization. A comprehensive evaluation of mechanical properties and porosity showed that at a BG content of 30 wt%, the composite scaffold achieved an optimal balance of properties, possessing an ideal porous structure while maintaining sufficient mechanical strength.

Furthermore, in vitro mineralization experiments in simulated body fluid (SBF) confirmed that the composite scaffolds exhibited good in vitro bioactivity, showing a positive correlation between the mineralization time and the formation of hydroxyapatite (HA). More importantly, in vitro cytological evaluations further verified the excellent biocompatibility of the material. The early adhesion of MC3T3-E1 cells demonstrated that the incorporation of BG effectively promoted cell adhesion and cytoskeletal development compared to pure PCL, with all BG-containing groups showing significantly higher fluorescence intensities than the Pure PCL group.

In summary, this research provides an experimental basis and theoretical reference for the design and fabrication of high-performance 3D-printed bone repair scaffolds. Particularly, the PCL/30BG composite scaffold stands out as a highly promising bone graft substitute, laying a solid materials science foundation for future clinical application studies.

We acknowledge several limitations in the present study. Although the formation of hydroxyapatite can be partially evidenced by SEM characterization, XRD patterns, and cellular activity assays, the direct chemical confirmation of biomimetic hydroxyapatite deposition via post-immersion FTIR was not performed due to the limited sample size and the lengthy SBF incubation period. Furthermore, the complex 3D structure of the scaffolds hindered the acquisition of clear bright-field images and cross-sectional morphology. We will pay close attention to these issues and designate them as the focus of our future research.

## Figures and Tables

**Table 1:** Composition of the bio-ink for composite scaffolds.

Sample	PCL (g)	BG (g)	CHCl_3_ (mL)	DMSO (mL)
Pure PCL	2	0	6.8	0.2
PCL/10%BG	2	0.222	6.8	0.2
PCL/20%BG	2	0.500	6.8	0.2
PCL/30%BG	2	0.857	6.8	0.2
PCL/40%BG	2	1.333	6.8	0.2

**Table 2:** DSC thermal parameters of PCL/BG composite scaffolds.

Sample	Tm (°C)	ΔHm (J/g)	Xc (%)
Pure PCL	72.83	71.80	51.47
PCL/10%BG	70.96	57.94	46.15
PCL/20%BG	69.64	51.21	45.89
PCL/30%BG	67.91	42.40	43.42
PCL/40%BG	65.76	30.33	36.23

## Data Availability

The original contributions presented in this study are included in the article. Further inquiries can be directed to the corresponding author.
